# Revealing the Unique Themes in Parent–Child Shared Book Reading Behaviors: A Systematic Review of Chinese and English Research 2005–2024

**DOI:** 10.3390/bs16040581

**Published:** 2026-04-13

**Authors:** Junnan Zhou, Jingyi Lei, Shuang Chao, Chenyi Zhang

**Affiliations:** 1 Faculty of Education, Tianjin Normal University, Tianjin 300387, China; leijingyi2026@stu.tjnu.edu.cn (J.L.); cshuang@stu.tjnu.edu.cn (S.C.); 2 Department of Early Childhood and Elementary Education, College of Education and Human Development, Georgia State University, Atlanta, GA 30303, USA; czhang15@gsu.edu

**Keywords:** parent–child shared book reading, reading behavior, Chinese and English contexts

## Abstract

This study provides a systematic review of research hotspots and trends in the field of parent–child reading, covering the period from 2005 to 2024, based on data retrieved from the China National Knowledge Infrastructure (CNKI) and the Web of Science (WOS). The results indicate that both Chinese- and English-context research on parent–child reading focus on the family literacy environment, the impact of parent–child reading on child development, social support systems, and educational equity. Chinese research places greater emphasis on family reading, family–kindergarten collaboration, and father involvement. This research mainly examines parental guidance strategies and pays particular attention to current practices, especially in rural areas. It highlights the role of fathers in reading, with picture books being the most commonly used reading materials. In contrast, English-context research focuses more on language development and early literacy, with particular emphasis on the development of children’s literacy skills and school readiness. Greater attention is also given to multicultural and minority groups, the role of mothers in reading is more frequently emphasized, and the reading materials are predominantly storybooks and wordless books. Research in both Chinese and English contexts reveals that parent–child reading interactions serve as a channel for the transmission of cultural values, leading to distinct developmental priorities for children. These differences profoundly reflect the systematic influence of sociocultural logics on parental reading behaviors and related research. This analysis provides an empirical foundation for future international collaboration in cross-cultural research.

## 1. Introduction

Parent–child shared reading is defined as a playful, interactive engagement with literature within a relaxed and emotionally supportive environment (Ji, 2006). Often utilized as a primary strategy for promoting early literacy, this approach encourages parents and children to move beyond the literal text through open-ended questioning and extrinsic conversation (Hassinger et al., 2016). It is characterized by the joint construction of meaning, whether the adult reads to the child, the child reads to the adult, or both engage in the text together. This dynamic process often involves interactive play and conversation, creating a socio-emotional context for learning (Levy & Hall, 2021). By facilitating a deeper understanding of narrative structures while simultaneously cultivating reading strategies and positive habits, parent–child shared book reading establishes a foundation for lifelong literacy development (C. M. Zhu & Zhou, 2006). This is particularly important for young children in early childhood when early literacy skills are gradually emerging. Beyond its cognitive benefits, parent–child shared book reading serves as a catalyst for academic curiosity, the cultivation of adaptive learning dispositions, and the development of positive character traits (Y. Li et al., 2024). It also strengthens the parent–child relationship through consistent language and emotional exchange (Tian, 2023). From a broader sociological perspective, parent–child book reading should be recognized as a culturally significant practice that contributes profoundly to a child’s socialization (Stangeland et al., 2023). Ultimately, parent–child book reading contributes to a quality home literacy environment, fostering children’s socio-emotional development (Rose et al., 2018).

While early literacy research has extensively documented the contributions of parent–child shared reading to developmental outcomes, these investigations have historically been situated within English language contexts, such as the United States (e.g., Bus et al., 1995; Mol et al., 2008). It is only recently that scholars have begun to systematically examine the impact of shared book reading within the Chinese language context (Zhou, 2023). Current Chinese scholarship primarily interrogates the practices, values, and strategies associated with the book-reading activity (Xu et al., 2022), drawing on diverse disciplinary perspectives such as clinical counseling, psychology, and educational science (Xie et al., 2018). However, despite this growing theoretical foundation, the existing literature in China remains predominantly qualitative, with a notable dearth of robust quantitative analysis (Xing, 2020). In contrast, research on parent–child reading in English-speaking contexts (e.g., the United States, the United Kingdom, Mexico, Australia, etc.) not only recognizes the value of parent–child reading but also focuses on studying different reading approaches of shared book reading (Treiman et al., 2018; Bindman et al., 2014). Researchers have also linked children’s book-reading experiences with parents to children’s early literacy skills, with consideration of ecological factors such as children’s genders, family income levels, and ethnic backgrounds (Chan et al., 2023; Cutler, 2023; Rie et al., 2020). Researchers emphasize experimental interventions in parent–child reading and employ quantitative methods for related research (Goldfeld et al., 2011).

The discrepancies between literacy research in Chinese and English language contexts may reflect the different theoretical conceptualizations that reflect unique cultural values. From a socio-cultural perspective (Vygotsky, 1978), early literacy activities, such as shared book reading, support both children’s cognitive growth and cultural enculturation (Vygotsky, 1978), which are shaped by the normative expectations of a specific social context. For example, during book reading, children’s learning is a socially situated process wherein knowledge construction is mediated not only by a “More Knowledgeable Other” (MKO), such as a parent, but also by cultural tools like storybooks. While reading behaviors may appear superficially similar across cultures, underlying foundations profoundly influence parental objectives and the subsequent interpretation of a text’s significance (Chao, 2000).

In the Chinese context, parent–child shared reading is often understood as a parenting practice closely tied to parent–child relationship building (Gui & Zhu, 2021), family harmony (Q. Zhao & Xu, 2014), and culturally embedded expectations of parental responsibility; these expectations have also been discussed in relation to traditional Confucian family ethics such as filial piety and family-oriented moral cultivation (Q. Q. Zhang, 2015). In 2023, China launched the national Family Parent–Child Reading Campaign of “Fragrance of Books Spreads to Every Household”, which emphasized that parent–child reading could enhance parents’ capacity for scientific parenting and boost children’s interest in and competence for reading. In 2025, China promulgated the Regulations on the Promotion of National Reading, which also stipulated that parents of minors should play an exemplary role through their words and deeds, carry out feasible family reading and parent–child reading activities, and help minors develop good reading habits. In other words, parents should provide home learning activities to fulfill parenting responsibilities (Du, 2021). There is an explicit purpose of socialization of cultural values in parent–child book reading in the Chinese context, in that parents focus on introducing social norms, moral virtues, and family-centered role expectations to children through book reading (J. Li et al., 2014). Consequently, the parent–child interaction in book reading is often characterized by structured guidance and didactic instruction, ensuring that children grasp specific moral lessons and conform to social expectations and standards (Luo et al., 2013). In contrast, within the individualistic contexts of many Western, English-speaking countries, shared book reading is conceptualized as a mechanism for fostering children’s autonomy, creative expression, and cognitive exploration. Rather than prioritizing moral value transmission, Western parents tend to emphasize child-centered engagement, reading development, and emotional well-being. These interactions often utilize open-ended questioning and collaborative narration to promote children’s independent thinking and personal agency (Miller et al., 2012; Tamis-LeMonda et al., 2019).

These divergent cultural priorities—socialization and virtue in the Chinese context versus intellectual autonomy in the Western context—directly influence the trajectory of shared reading research. Some cross-cultural studies illustrate this divide. For example, research showed that while American mothers often prioritize intellectual stimulation and the expression of positive affect, Taiwanese mothers are more likely to utilize the text to discuss the cultivation of virtues and the regulation of negative emotions (J. Li et al., 2014). This suggests that while parents, internationally, recognize the developmental value of book reading (Tang, 2023), their book-reading behaviors are driven by different purposes. Nearly two decades ago, picture books solidified their position as an established best-selling genre of children’s literature in mainland China and emerged as a pivotal medium for parent–child shared reading. In the years that followed (Kong et al., 2024), a succession of policies to advance early reading have been enacted, and international academic exchanges in this research domain have developed incrementally. This study aims to compare the thematic orientations of parent–child shared book reading research in Chinese- and English-language contexts for advancing the global literacy research collaboration. Recognizing these thematic trajectories in the past 20 years is vital for international collaborators. It supports mutual understanding about research topics that are valued within respective cultural contexts. Comparative analysis facilitates semantic alignment, ensuring that researchers from different language contexts are operating from a shared conceptual understanding. For example, while a Western collaborator may interpret a parent’s structured, factual questioning as “restrictive”, a Chinese counterpart may recognize it as a deliberate strategy for “moral scaffolding”. By identifying these thematic discrepancies, researchers can develop culturally sensitive methodologies that recognize the localized norms inherent in home literacy practices. In addition, comparing these research landscapes allows for an effective integration of diverse scientific traditions in collaboration. The robust experimental and quantitative frameworks frequently used in English language contexts can be enriched by the deep, qualitative, and value-driven insights characteristic of Chinese literacy research. This synthesis provides a more holistic understanding of child development, one that accounts for both the universal cognitive mechanisms of literacy and the particular cultural tools that mediate learning (Vygotsky, 1978).

In this research, CiteSpace is used to systematically analyze parent–child reading research; the analyses aim to reveal the changing themes of parent–child book reading research in the past 20 years.

This research proposes the following questions:

1. What are the unique themes of parent–child reading research in the Chinese and English context?
2. What are the trends of parent–child reading research in the Chinese and English context?

## 2. Method

### 2.1. Data Collection

**Figure 1:**
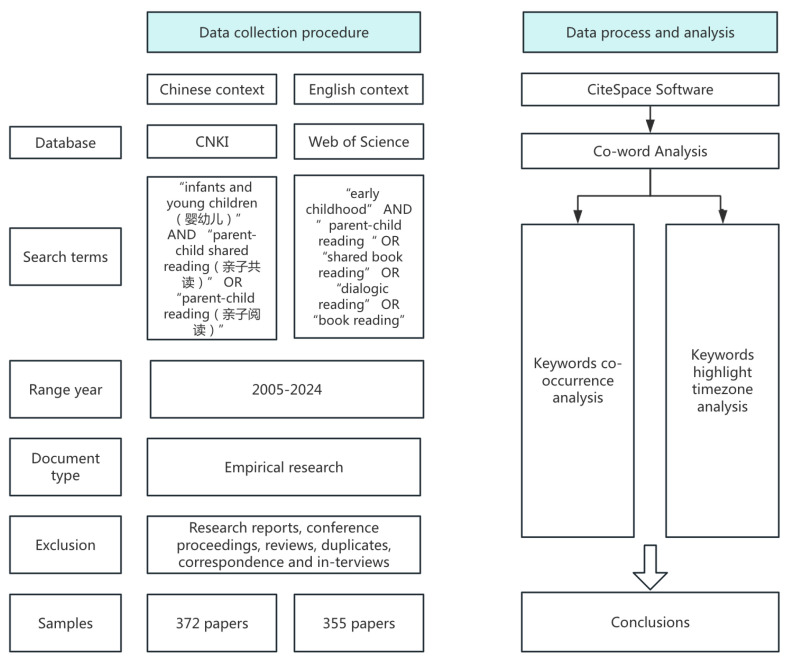
Integrated analysis framework.

A comprehensive literature search was conducted in the China National Knowledge Infrastructure (CNKI) database and Web of Science (WOS) database spanning the period from 2005 to 2024 (the deadline for data collection is 31 December 2024). CNKI is the largest and most authoritative Chinese academic resource integration platform in China, hosting academic peer-reviewed scholarly publications in Chinese. WOS is the most authoritative multidisciplinary research database platform, primarily hosting academic publications in English. Figure 1 illustrates the procedure of the literature search. Only peer-reviewed journal publications were included in the search.

To ensure a high degree of methodological transparency and rigor, the literature selection process for this study was conducted in three phases. As shown in Figure 1, in the initial phase, a comprehensive full-text search strategy was employed across the CNKI and WOS databases to maximize coverage and minimize the risk of omitting seminal works. For the Chinese language context, the search utilized Boolean operators with the terms “infants and young children (婴幼儿)” AND “parent–child shared reading (亲子共读)” OR “parent–child reading (亲子阅读)”, yielding an initial pool of 9404 documents. For the English-language context, the query targeted “early childhood” AND “parent–child reading” OR “shared book reading” OR “dialogic reading” OR “book reading”, which, after applying the “Full Text” filter, identified 2518 documents. This wide-net approach, using full-text match of keywords, while significantly increasing the initial volume of records, guarantees that no nuanced or emerging studies were overlooked in the foundational stage of the review.

Following the initial retrieval, the second phase involved an independent screening of titles and abstracts by two researchers to ensure strict thematic alignment with parent–child shared reading behaviors. This phase focused on isolating studies where shared reading was a central variable in empirical studies, resulting in a narrowed pool of 2794 Chinese and 943 English-language records. To maintain high inter-rater reliability, the researchers cross-referenced their findings and resolved any discrepancies regarding inclusion through deliberative discussion and consensus-building. This verification step ensured that the dataset remained focused on core research themes while accounting for the cultural nuances inherent in both language contexts.

In the final phase, rigorous exclusion criteria were applied to refine the sample into a collection of empirical studies suitable for CiteSpace analysis. Non-empirical works, including conceptual articles, non-peer-reviewed essays, and technical reports, were eliminated, alongside non-data-driven scholarly works such as conference presentations, book reviews, and correspondence. Additionally, duplicate entries were removed to ensure data integrity. In this phase, to ensure the ecological validity and cultural specificity of the comparative analysis, a secondary layer of refinement was applied to filter out studies that did not align with the primary linguistic and cultural contexts of their respective databases. Specifically, Chinese-language empirical studies within the CNKI database examining non-Chinese populations, such as American or Western cohorts, were systematically excluded. Conversely, English-language studies from the WOS database that focused specifically on Chinese or Chinese-immigrant families were likewise removed from the article pool. This decision aimed to isolate the idiosyncratic research trajectories and culturally embedded priorities inherent to each native context. By restricting the data to research conducted within and for each respective language context, the study minimizes the potential for the confounding effects of acculturation often observed in international studies. This approach allowed for a clear extraction of native research hotspots in Chinese and English language contexts, revealing how academic research naturally prioritizes different variables in respective societies. Narrowing the scope also supports clear CiteSpace visualizations revealing distinct and robust keyword clusters.

This systematic review resulted in a final dataset of 372 valid articles in Chinese from CNKI and 355 articles in English from WOS. These 727 documents constitute the definitive empirical foundation for the subsequent bibliometric and cross-cultural comparative analysis, providing a balanced and representative dataset of the global research landscape from 2005 to 2024.

### 2.2. Data Analysis

To address the proposed research questions, this study utilizes CiteSpace (version 6.3.R3), a specialized bibliometric visualization tool designed to map the structural and temporal dynamics of scientific knowledge domains (Chen et al., 2015). By synthesizing large-scale bibliographic data, CiteSpace enables the identification of the “intellectual base” and the “research front” of a discipline. In this study, the software serves as the primary methodological framework for visualizing the intrinsic connections within the literature, allowing for the rapid identification of research hotspots and shifting paradigms across the 2005–2024 period.

To identify the unique themes within the Chinese and English contexts in Research Question 1, we constructed keyword co-occurrence networks and cluster mappings. These visualizations provide a topological representation of the field, where high-frequency keywords and high-centrality nodes highlight the core thematic “hotspots” prioritized by researchers in each linguistic tradition. By clustering these keywords, the study distills complex bibliographic data into coherent thematic categories, such as the English focus on “dialogic reading” and “socio-emotional outcomes” versus the Chinese emphasis on “academic readiness” and “cultural transmission”. In this way, the prioritized themes of research in each language context can be revealed.

To illustrate the developmental trends of book reading research in Research Question 2, a longitudinal analysis is conducted using timeline visualizations and burst detection algorithms. The timeline diagrams provide a diachronic view of how specific research clusters have emerged, matured, or faded over the past two decades, illustrating the evolutionary paths of the field. Furthermore, “citation bursts” are utilized to identify surging areas of interest that signal upcoming research frontiers. This quantitative mapping is subsequently complemented by a systematic content analysis, allowing for a refined synthesis of the specific research trajectories. By comparing the findings from the two language contexts, the study explores the underlying influence of cultural traditions and educational systems, offering a robust theoretical foundation for future cross-cultural collaboration and communication.

## 3. Results

### 3.1. Publication Volume and Trends

**Figure 2:**
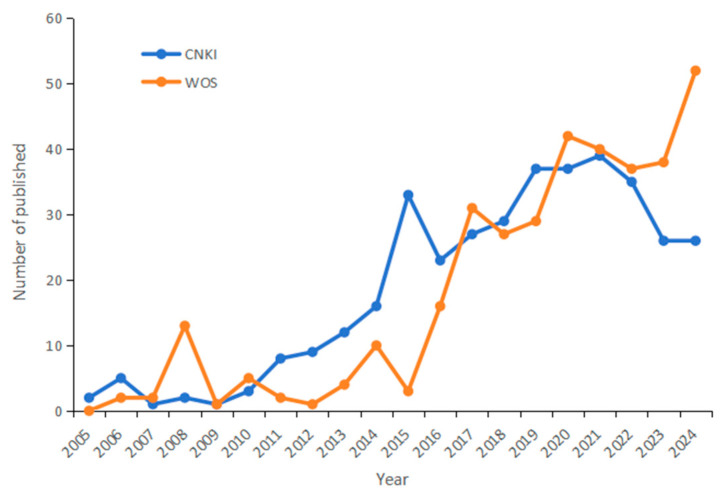
Annual publication volume of parent–child reading research in the Chinese and English contexts from 2005 to 2024.

Figure 2 shows the number of publications on CNKI and WOS from 2005 to 2024. Overall, there is an increasing trend in the number of articles published. Temporally, the trend is broadly consistent across the two platforms. The period between 2005 and 2014 marks the nascent stage of parent–child reading research, with a relatively small number of publications, indicating limited engagement.

After 2015, there was a significant increase in the number of articles published. This may be related to the promulgation of relevant policies in the Chinese and English contexts. In China, “national reading” has been included in “the Government Work Report” for 12 consecutive years since 2014. In 2015, the Ministry of Education and two other government agencies jointly issued a policy document advocating “families reading a good book together” and promoting the establishment of parent open days in libraries. And “Opinions on the Promotion of National Reading”, issued in 2020, further strengthened this policy orientation, reflecting the country’s continued attention to the promotion of national reading.

Nations of English-speaking contexts have also advanced sustained reading promotion initiatives. The Finnish Reading Center launched the “Gift of Reading for Children” program, which aims to provide children with early reading opportunities. The UK’s “Bookstart” program was dedicated to ensuring that every British child benefits from early reading, with the fundamental principle of fostering a lifelong love of reading through the enjoyment of reading. Australia promulgated the National Early Language and Literacy Strategy, which emphasizes the role of parents in early language learning. These initiatives indicate that different countries attach great importance to the development of children’s early reading ability.

Overall, parent–child reading research in both Chinese and English contexts has been influenced by national reading policies. Research in China has exhibited a slight downward trend since 2021; however, with the national policy-driven advancement of reading initiatives and the deepening of international academic exchanges, it is also poised to show a broad and promising prospect for development.

### 3.2. The Unique Themes Reflected by Research Hotspots

#### 3.2.1. Keywords Co-Occurrence

Keyword co-occurrence analysis reveals terms that appear frequently in a given time period, highlighting hot topics in the research field (H. Li et al., 2024). The map consists of lines and nodes, with larger nodes indicating higher co-occurrence frequencies of the corresponding term with other keywords. The lines denote the connections between nodes.

**Figure 3:**
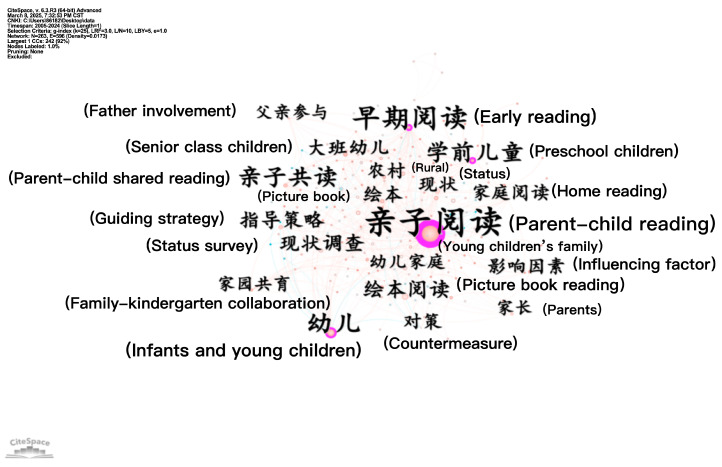
Co-occurrence map of keywords in parent–child reading research in China.

Figure 3 shows a network of 263 nodes and 596 connections (density = 0.0173). High-frequency keywords in China include “parent–child reading”, “infants and young children”, “early reading”, “picture book reading”, “guiding strategies”, “status quo”, etc. The appearance of “father’s involvement” and “rural” indicates that parent–child reading research has gradually expanded from focusing on parent–child reading itself to focusing on different regions and participants.

**Figure 4:**
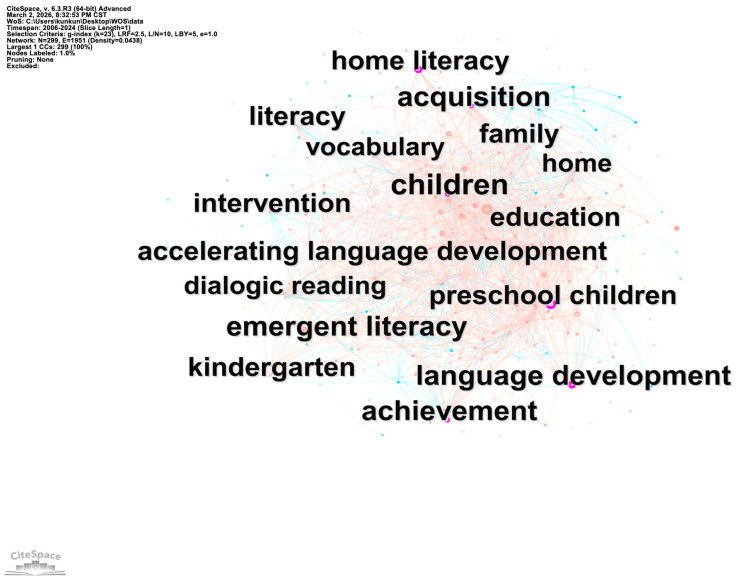
Co-occurrence map of keywords in parent–child reading research in the English context.

Figure 4 shows a network with 299 nodes and 1951 connections (density = 0.0438). In the English context, the keywords that appear with high frequency include “language”, “acquisition”, “intervention”, “vocabulary”, “emergent literacy”, and “family”, etc. The prominence of the keywords “language”, “vocabulary”, and “intervention” indicates that English-context research has focused mainly on children’s language literacy and family literacy, emphasizing children’s vocabulary development, intervention research, and the impact of family involvement.

In summary, the number of nodes in research across Chinese and English contexts is comparable, yet the number of connections in English-context research is considerably higher than that in Chinese-context research. Chinese-context research places strong emphasis on guiding parental engagement and the content selection of parent–child shared reading, whereas English-context research has paid more attention to the development of children’s language and emerging literacy skills in parent–child reading, as well as family participation.

Centrality is a quantitative metric that quantifies the probability of a specific node lying on the shortest path between any two nodes in a network. This metric further reflects the structural role of a node in mediating interconnections with other network nodes (Dang et al., 2021). As shown in Table 1, keywords with centrality greater than a value of 0.1 possess greater academic significance and carry more structural weight in the research network.

In China, the top three keywords in terms of centrality are “parent–child reading”, “young Children”, and “early reading”, which indicates that they are closely related to each other. Parent–child reading is an important form of early reading; young children are the subject of parent–child reading and early reading, and early reading provides content and goal orientation for parent–child reading.

The top three keywords in the English context in order of centrality are “intervention”, “acquisition”, and “family”. The focus is on the development of vocabulary comprehension, phonological awareness, and oral language in young children through parent–child reading. “Intervention” suggests that a dialogic reading intervention for parents and children, primarily through an experimental approach, is effective in promoting early language and literacy development in young children. Furthermore, attachment has also emerged as one of the highest centrality keywords. Parent–child reading transcends being merely a cognitive activity—it is a profound socio-emotional interaction. This makes it an ideal setting for building and strengthening secure attachment bonds. Here, attachment refers to a lasting emotional connection forged through physical closeness, emotional responsiveness, and shared attention. It serves as both the foundation for quality parent–child reading and a key emotional outcome.

Therefore, we can conclude that Chinese research on parent–child reading emphasizes the importance of early reading and early childhood development, whereas English-context research emphasizes intervention in the reading process and the influence of the family. At the same time, the diversity of family systems and differences in social environment on the early development of children were also considered.

#### 3.2.2. Keyword Cluster

Keyword clustering was conducted to identify the major thematic structures within the field of parent–child reading. By analyzing keyword co-occurrence relationships, semantically related and frequently co-occurring terms were automatically grouped into distinct clusters. Each cluster represents a relatively independent subdomain, which helps reveal the knowledge structure of parent–child reading research as well as the composition and interconnections of its core themes. In this way, the clustering analysis directly supports the study’s aim of comparing the thematic focus and research patterns across Chinese and English contexts. The keyword clustering analysis categorizes knowledge domains to provide deeper insight into research hotspots. A *Q* value (modularity) greater than 0.3 indicates significant clustering, an *S* value (weighted average profile coefficient) greater than 0.5 indicates reasonable clustering, and an *S* value greater than 0.7 suggests that the clustering is efficient and compelling.

**Figure 5:**
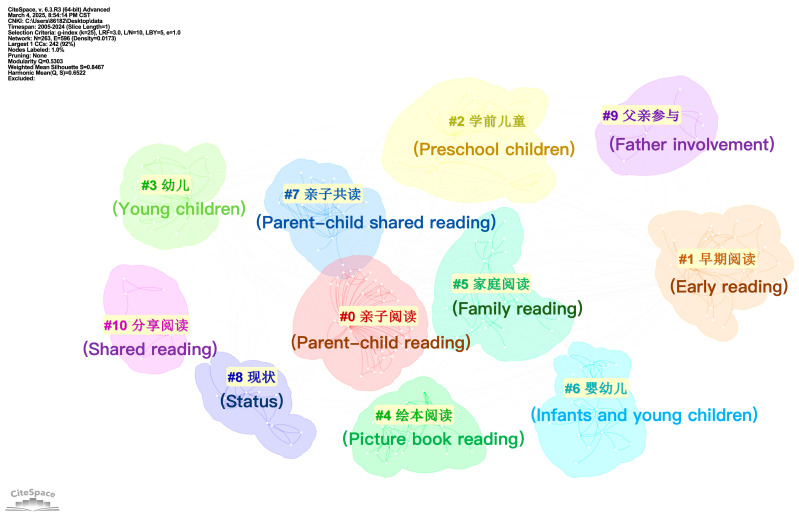
Cluster map of keywords in parent–child reading research in China.

Figure 5 shows that the CNKI analysis identified 11 clusters with a modularity of *Q* = 0.5303 and an average profile coefficient of *S* = 0.8467, indicating significant and efficient clustering. The main cluster labels were “parent–child reading”, “early reading”, “preschool children”, “picture book reading”, “family reading”, “infants and young children”, “status”, and “father involvement”. Chinese researchers have explored the role of picture books in parent–child reading and emphasized the positive effects. Parent–child reading can help young children enrich their vocabulary, promote knowledge construction, and enhance their language expression and thinking skills. However, there is still room for further research on the extent of parents’ participation in parent–child reading and the status quo in different geographical areas.

**Figure 6:**
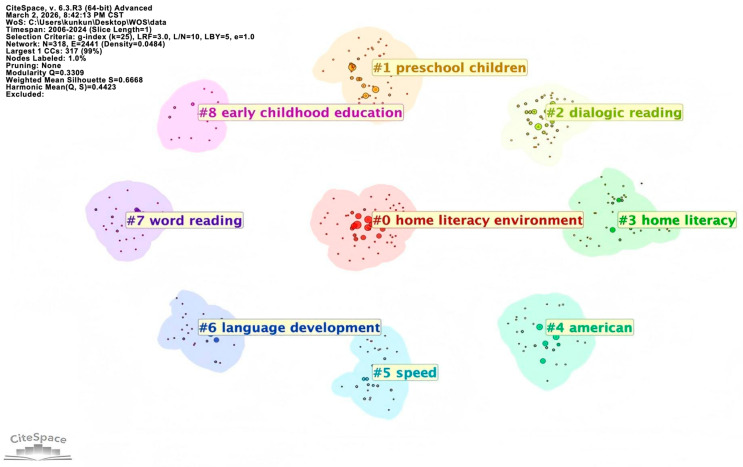
Cluster map of keywords in parent–child reading research in the English context.

Nine clusters were identified through WOS analysis (Figure 6), with a modularity *Q* of 0.3309 and an average profile coefficient *S* of 0.6668, indicating valid clustering. The main cluster labels included “home literacy environment”, “preschool children”, “dialogic reading”, “home literacy”, “language development”, and so on. This reflects that English-context researchers have focused on young children’s reading skills and interventions, emphasizing the influence of multiple factors, including family socioeconomic status, the home literacy environment, and risk factor analysis, on young children’s development. Paakkari et al. (2024) explored the favorable and unfavorable family environmental factors that affect the development of young children’s critical reading abilities, among which parents’ attitudes and abilities play an important role. Niklas et al. (2020) hold the view that the family literacy environment plays a mediating role in the influence of parents’ reading attitudes and children’s language abilities. Tanji and Inoue (2023) found that family access to literacy resources is associated with children’s early reading abilities. Linberg et al. (2020) show that the quality of shared picture book reading with infants and early reading initiation is associated with children’s vocabulary size.

### 3.3. The Trends Reflected by Timeline and the Strongest Citation Bursts of Keywords

#### 3.3.1. Timeline of Keywords

**Figure 7:**
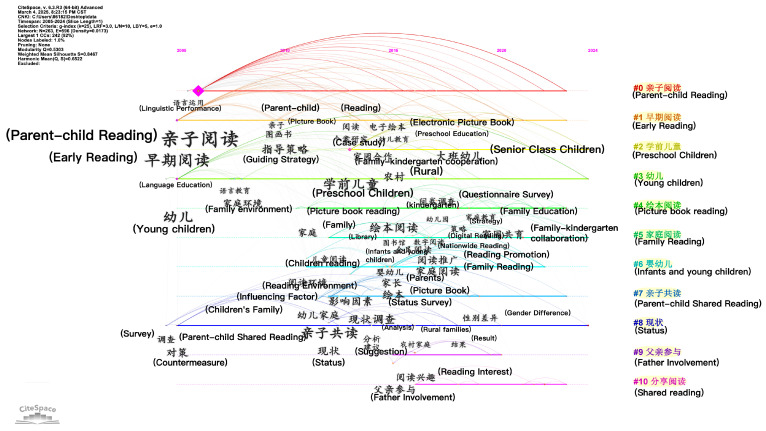
Timeline map of keywords in Chinese parent–child reading research.

To address the limitations of keyword co-occurrence and clustering analysis, timeline-based keyword mapping was employed to identify core research themes across temporal dimensions. The results are shown in Figure 7.

The terms “parent–child reading”, “early reading”, “young child”, “countermeasures”, and “linguistic performance” emerged earlier in the CNKI database, reflecting that Chinese research during this stage prioritized early parent–child reading, with a focus on practical methods and guidance strategies, as well as the characteristics of language use by mothers in parent–child reading (Ji, 2006; Zhou & Zhu, 2006). The subsequent emergence of keywords such as “family –kindergarten collaboration”, “rural areas”, “picture book reading”, “status survey”, “father’s involvement”, and “reading promotion” highlights a shift toward diversified research directions with stronger practical and applied value, demonstrating expanded breadth and depth. The concept of reading is understood from small to big, from individual to collective, and from close family environment to social environment, and it is committed to creating a “reading for all” atmosphere. Chinese research has evolved from emphasizing the importance of reading and guidance strategies to investigating reading materials, influencing factors, regional disparities, and promotion frameworks. Future research may further concentrate on home–kindergarten coeducation and nationwide reading promotion initiatives.

**Figure 8:**
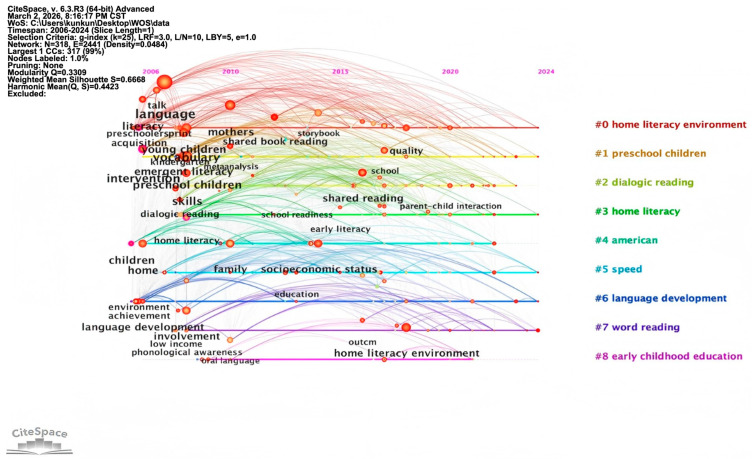
Timeline map of keywords in the English context, parent–child reading research.

Figure 8 reveals that terms such as “language”, “emergent literacy”, “mothers”, “intervention”, and “low income” appeared in early research within the WOS database, indicating that English-context researchers initially focused on early literacy skills, language development, the role of mothers in reading, and the impact of socioeconomic factors on reading practices, with an emphasis on supporting family reading through targeted interventions (Brown et al., 2018; Lingwood et al., 2020). Subsequent research has explored multidimensional influencing factors (Shen & Del Tufo, 2022), parent–child interaction patterns (Jacob et al., 2024), and the development of children’s language and social–emotional skills (Kim & Yim, 2024; Loredana et al., 2024). This evolution reflects an expansion of research from basic literacy interventions to more complex analyses of family dynamics and children’s cognitive development.

#### 3.3.2. Strongest Citation Bursts of Keywords

By applying keyword burst detection analysis, we trace the evolution of research hotspots, offering insights into emerging trends and potential future directions in parent–child reading. The analysis identified the top 15 keywords with the strongest citation bursts; red indicates the length of the observed term burst, whereas azure indicates its duration (H. Li & Tinmaz, 2024).

**Figure 9:**
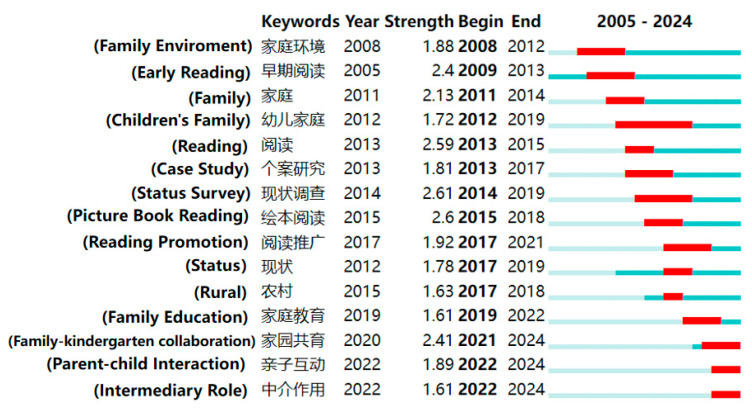
Top 15 keywords with the strongest citation bursts in Chinese parent–child reading research. Note. Red indicates the length of the strongest citation bursts, whereas azure indicates the duration of the keywords.

Figure 9 illustrates the 15 emergent terms in the CNKI. The most intense term is “status survey” (2014–2019), during which many researchers investigated the current status of parent–child reading, focusing on exploring the current state of parental involvement in parent–child reading and the issues that arise in the reading guidance process, and helping parents use the correct reading guidance methods (J. Wang, 2015; Fan, 2018; F. Wang & Liu, 2019). “Children’s family” is the longest-lasting buzzterm (2012–2019), indicating its lasting attention and depth of research. The latest buzzwords are “parent–child interaction” and “intermediary role”. As an important way of reading, parent–child reading can not only promote the development of children’s reading abilities but also enhance the feelings between parents and children and improve the comprehensive quality of parents (Tian, 2023). Research has shown that the family literacy environment plays a partial intermediary role between family socioeconomic status and the development of children’s ability (Yao et al., 2022).

**Figure 10:**
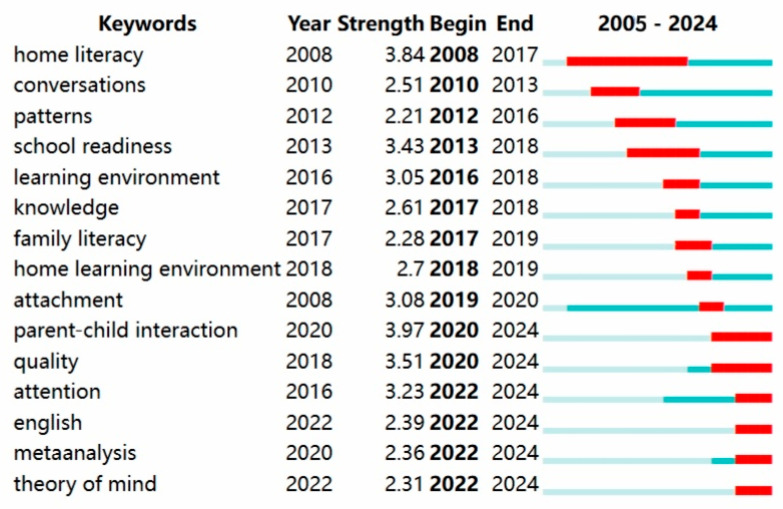
Top 15 keywords with the strongest citation bursts in English parent–child reading research. Note. Red indicates the length of the strongest citation bursts, whereas azure indicates the duration of the keywords.

Figure 10 shows the emergent keywords in the English context, where “home literacy” has the highest emergent intensity. In terms of the duration of keyword emergence, “home literacy” also had the longest emergence period, from 2008 to 2017. The home learning environment (HLE), as a key factor influencing children’s long-term development, serves as a crucial backdrop for the acquisition of essential developmental skills (Niklas & Schneider, 2013). Primary caregivers can support children’s learning through shared reading in daily interactions, thereby influencing their socio-emotional development (Niklas et al., 2021). In addition, different socio-economic backgrounds, family reading and writing environments (shared books, quality of verbal interaction, and parents’ “mental state”), and mother tongue will also have an impact on children’s language and theoretical thinking (Ebert et al., 2020). Recently, words such as “theory of mind” have emerged, which show that English-context researchers have paid more attention to exploring the influencing factors of parent–child reading and its influence on children’s learning abilities and have emphasized improvements in reading quality and the cultivation of children’s comprehensive learning abilities (Gavora, 2022; Bergström et al., 2024).

## 4. Discussion

This research compares and analyzes the behavioral logic of parent–child reading research in the Chinese and English contexts and reveals the underlying reasons behind it. It mainly covers the unique themes and trends.

### 4.1. The Unique Themes

The findings of the analysis of co-occurrence and centrality indicate that interconnections among research themes in English-context studies are significantly closer than those in Chinese-context counterparts. Chinese-context research themes are relatively homogeneous, with the majority focusing on reading behavior per se and centering on the construct of family responsibility. In the Chinese context, intergenerational reading serves as a vehicle for passing down fine family traditions and culture, transforming memories and ethical concepts in language into practical life experiences. Guidance and inheritance play a significant role in Chinese families (X. X. Wang & Chi, 2019). By contrast, “intervention” emerges as the keyword with the highest centrality in English-context research, which reflects a more nuanced and detailed dissection of the relevant influencing factors.

#### 4.1.1. From Fathers and Mothers as Research Participants

“Father” emerges as a keyword in Chinese research. Against the backdrop of China’s enactment of the Family Education Promotion Law and its emphasized commitment to gender equality, researchers have turned their attention to the current status and value of fathers’ involvement in childcare. Therefore, researchers have begun to pay attention to the role of fathers in family education and emphasize the importance of fathers’ participation in early childhood education (J. Liu, 2025). Chinese research pays more attention to the participation of fathers in parent–child reading, especially how fathers can establish an emotional connection with their children through reading and promote their children’s overall development. Chinese research has focused on exploring the current status of fathers’ involvement (Shi & Cao, 2019; Y. Zhao, 2020), including the behavioral logic of fathers’ absence in parent–child reading (Y. H. Liu & Liu, 2021), interaction patterns between fathers and young children, and how these interaction patterns influence children’s behavioral performance (L. Liu et al., 2019), and the impact of fathers’ parenting involvement behavior on children’s interest in reading (X. Q. Zhu, 2020). Empirical evidence has indicated that fathers’ participation in parent–child shared reading not only fosters children’s positive reading habits but also effectively strengthens parent–child bonds and cultivates a harmonious family climate (Q. X. Zhang, 2017).

In English-context research, the family structure is dominated by the nuclear family; the father’s role in parent–child shared reading has not been explicitly highlighted or differentiated. Even more attention has been given to the participation of mothers in parent–child reading. For example, mothers’ personal engagement behavior during mother–child reading, particularly concerning how they use strategies of mental state language and cognitive state language, relates to children’s development of false belief understanding (Tompkins, 2015), and their potential dynamic influence on children’s theory of mind (H. Liu et al., 2024). This type of linguistic interaction behavior indirectly influences the construction of children’s theory of mind abilities by shaping their cognition. Some researchers have reported that different mothers adopt different reading styles, which also have different longitudinal predictive abilities for children’s literacy (Caspe, 2009). In addition, when the function of the nuclear family is missing, society will provide alternative support, which reflects the characteristic that individuals can get support from multiple social networks (Levy & Hall, 2021).

#### 4.1.2. From Different Groups as Research Participants

Research in both Chinese and English contexts, both examining parent–child reading and social equity, fundamentally explores how specific sociocultural contexts influence psychological and behavioral processes within the family. Chinese research focuses on specific groups of parent–child reading in rural areas, often on the basis of regional surveys. China is currently advancing the vigorous development of rural education to bridge the urban-rural gap in educational access and quality. A review of the literature reveals that there is more research on the current situation of early reading, educational characteristics, and influencing factors of rural left-behind young children. Chinese scholars, through urban-rural comparisons, have focused on analyzing differences in family cognitive and behavioral resources systematically shaped by socioeconomic status disparities. Investigations have revealed a severe lack of early reading practices within the families of rural left-behind children, a gap that necessitates parents establishing a scientifically sound understanding of early reading, as well as enhancing their awareness of its developmental importance and their capacity to implement evidence-based early reading guidance (H. X. Guo, 2013). In terms of influencing factors, individual, environmental, and social factors all affect the reading status of rural left-behind young children (Qiang, 2021). The urban-rural gap manifests not only in material resources but more profoundly in disparities among parents regarding knowledge schemas for early education, patterns of time investment, and the quality of language interaction. Differing social environments in urban and rural areas shape parents’ educational concepts and behaviors toward early childhood education, resulting in disparities between urban and rural parents in parenting philosophies, interaction patterns, and strategy implementation (Yang, 2022). Owing to the imbalance of educational resources between urban and rural areas in China, the research is more focused on how to solve practical problems through practice, such as the promotion of parent–child reading in rural areas (Liang et al., 2015). Related research endeavors to explore how external support can alter parental parenting beliefs and daily practices to bridge gaps in early childhood cognitive stimulation. The core focus is to uncover and intervene in key psychological mechanisms that may contribute to inequality (X. Y. Guo, 2015; Z. Y. Zhang & Zhao, 2021). Chinese policies also focus on the behavior of parent–child reading in rural areas, imparting methods and techniques of parent–child reading to parents through forms such as parent schools, helping rural parents acquire effective reading knowledge and experience, and organizing various parent–child reading activities to create a good reading environment (H. X. Guo, 2013).

There were more large-scale cross-cultural comparisons in English-context research. The social structure of class reproduction makes the research focus more on “cultural capital” and “class solidification”, which reflects the social logic of individual competition and cultural reproduction (D. Wang et al., 2023). Children with multicultural backgrounds, such as African Americans, Latino children, and other specific ethnic groups, were research participants. African American mothers’ reading behaviors exert a profound influence on children’s academic performance by affecting mediating psychological variables such as language acquisition, learning interest, and self-efficacy (Harris & Rothstein, 2014). This reveals the psychological pathways linking parenting behaviors to children’s cognitive development. Furthermore, African American children’s reading and math scores are closely associated with their fathers’ level of home involvement, as paternal engagement provides children with diverse role models and problem-solving strategies (Baker, 2014). English-context policies promote the educational development of ethnic minorities through community-based family literacy programs, and research has paid more attention to the special needs of these groups. English-context policies support mother tongue education, stimulating families’ inherent and sustainable motivation to read. Parents can directly engage in reading and discussion with their children in their native language, enhancing their ability and confidence to participate in their children’s learning and boosting their sense of educational efficacy. English-context research similarly focuses on disadvantaged groups but emphasizes directly empowering families and communities through compensatory social support systems. Some researchers have found that the home literacy environment in rural children’s families was significantly associated with caregivers’ maternal education, income level, and history of reading difficulty, as well as children’s language abilities and gender (Myrtil et al., 2019). Research and programs targeting low-income, immigrant, and minority families (e.g., community shared reading) operate on the premise that providing accessible resources, modeling interactive strategies, and creating inclusive cultural environments enhance caregivers’ parenting efficacy. This increases children’s exposure to high-quality language input while strengthening their positive emotional connections to reading and self-concept. For instance, community programs designed for refugee children aim to mitigate the inhibitory effects of trauma on learning capacity through safe, supportive group interactions, thereby reshaping children’s attitudes toward reading and their behavioral responses to it (Hadfield et al., 2024).

### 4.2. The Trends

#### 4.2.1. “Status Survey” vs. “Intervention”

From the analysis of keywords with the strongest citation bursts, the keyword with the highest strength in Chinese research is “Status Survey”. Chinese research focuses on the analysis of localized behavior, aims to capture real problems in parent–child reading through questionnaires and interviews, and focuses on providing parents with practical guidance strategies, thus relying more on descriptive methods and emphasizing phenomenal generalization. In the Chinese context, parent–child reading behavior is centered around the emotional connection of family interaction while also carrying the mission of inheriting educational traditions. This behavioral logic determines that its research methods tend to summarize patterns from existing experiences. Chinese research pays more attention to picture books with rich illustrations and less text as the main material for parent–child reading. The research finds that as a favorite reading material for young children and an important medium for parent–child reading, picture books play an important role in promoting children’s healthy physical and mental development and cultivating parent–child emotions (Fan, 2018). In addition, parents’ choice of picture books has become a hot topic of concern for many researchers with respect to the characteristics of young children’s physical and mental development, the variety of materials, and the influence of reading materials on young children (S. Y. Liu et al., 2020). With the development of the digital age, the creation of electronic picture books has brought great convenience in reading, so the difference between electronic and paper picture book reading has also become a concern of researchers in helping parents choose reading methods and strategies suitable for young children. Chinese research is often deeply rooted in the belief that early literacy experiences lay the groundwork for future academic development and lifelong learning habits. Consequently, many Chinese educational programs and parenting guides advocate for daily reading routines and the active involvement of parents in their children’s reading activities (Yu, 2017).

In English-context research, the one with the highest strength is “parent–child interaction”. Influenced by the positivist tradition, researchers are more inclined to use controlled methods such as intervention experiments and longitudinal research methods to explore the specific elements of the reading process, highlighting the scientific exploration of individual development patterns behind reading behavior. Through the exploration of the theoretical mechanisms behind reading, English-context research has attempted to scientifically evaluate children’s individual development and to assess the impact of parent–child reading on children’s language and cognitive development. English-context research focused mainly on the interactive situation, including the interactive characteristics of the parent–child reading situation (Stein & Rosemberg, 2012) and the interactivity of using storybook apps (Aliagas & Margallo, 2017). Storybooks are the main materials for parent–child reading in the English-context research, which are characterized by more words and rich plots to develop young children’s language skills and reading comprehension. Some researchers have found significant differences in parent–child conversational interaction processes through comparisons between wordless and worded storybook reading, with more interactions occurring when reading wordless books (Petrie et al., 2023). In addition, English-context research has focused on the effect of storybook reading on children’s cognition. Rhymes in storybooks can help children memorize words (Read et al., 2014). In addition, mothers’ word explanations and story content expansion are also related to children’s vocabulary levels (Korat et al., 2018). This body of work often explores strategies such as dialogic reading, where parents ask questions, encourage predictions, and discuss the content of the text to deepen comprehension and critical thinking. Moreover, English-context studies frequently examine the impact of socioeconomic status, parental education levels, and cultural backgrounds on reading practices within the family. In terms of the social environment, English-context research often examines the broader societal influences on reading habits, such as access to libraries, community literacy programs, and the role of technology in children’s reading experiences (Hendrix et al., 2019). This approach indicates that while the family is a primary influence, external factors also significantly impact a child’s development.

#### 4.2.2. “Nationwide Reading Promotion” vs. “Personal Early Literacy”

Chinese and English-context policies have different emphases. Chinese education policy emphasizes cooperation between families and schools, and the degree of parental involvement in children’s education is regarded as a key factor. Consequently, Chinese scholarship places greater emphasis on reading duration, content selection, and the attainment of academic and moral readiness (J. Li et al., 2014; Luo et al., 2013; H. Wang, 2012). Chinese research highlights the importance of “parental guidance” and “family interaction”, revealing that in the micro-domain of parent–child reading, “family responsibility” is concretized into parents’ educational participation behaviors. Chinese parents play the role of “instructors” in reading, which can be seen as their embodiment of viewing children’s development as the core responsibility of the family. Secondly, due to intensified social competition and excessively high expectations for children, educators’ concepts tend to become utilitarian, and early reading exhibits a utilitarian trend. Here, utilitarianism manifests as an isomorphism between “social” and “family responsibility” (Zhai, 2009). In terms of cultural context, parents play an important role in family education in traditional Chinese Confucian culture. Chinese Confucian culture emphasizes individuals’ responsibilities towards the family and the collective, educational policies emphasize cultural inheritance, and communication between families and schools focuses on children’s collective adaptability. Picture books can help parents build good parent–child relationships with young children through the unique combination of pictures and texts. Parents emphasize knowledge transfer and habit formation during reading, enabling young children to grasp reading skills earlier, with their understanding of reading primarily being instrumental cognition. Chinese research focusing on parent–child reading behavior seeks to examine the current state of interactive parent–child reading practices across diverse regions and family contexts, with the ultimate goal of informing guidance strategies to promote reading on a national scale.

English-context policies emphasize individual development, close relationships, and inclusive services to ensure children’s equality at the starting line. They gravitate toward analyzing interactional styles and dialogic approaches and promote ethnic minority education through community-based family literacy programs while focusing on individual experiences within this equitable environment (Rodríguez et al., 2009). Meanwhile, English-context scholarship emphasizes the mainstream narrative of “reading for pleasure”, which aligns more closely with an individual’s psychological and emotional development (Cremin & Scholes, 2024). Influenced by individualistic culture, English-context research emphasizes children’s individual development and cultivation of early abilities, focusing on how to promote children’s language and cognitive development through textual reading. Parents encourage children to make their own choices and expressions, focusing on free dialogue and thought provocation, which can stimulate young children’s intrinsic reading motivation, critical thinking, and language creativity. Individualist culture emphasizes personal uniqueness and self-actualization, educational policies focus on multicultural education, and families and schools prioritize meeting children’s individualized needs. In parent–child reading, parents are more inclined to become reading facilitators, and reading is seen as a behavior that develops children’s personality, thinking, and emotions. The implementation of reading intervention research can improve children’s language skills and literacy (Korat et al., 2013). In addition, theoretical research has focused on mothers’ mental state language in reading to explore its influence on young children’s language development and to improve the quality of parent–child reading (Tompkins, 2015).

## 5. Conclusions

Parent–child reading research shows a significant diversification trend over the past two decades: the number of publications in the CNKI and WOS databases continues to grow, showing an overall upward trend. Chinese research on parent–child reading emphasizes the importance of early reading and early childhood development, and future research may further concentrate on home–kindergarten coeducation and nationwide reading promotion initiatives. English-context research emphasizes intervention in the reading process and the influence of the family while also acknowledging the impact of diverse family systems and varying social environments on children’s early development. The scope of research has expanded from basic literacy interventions to more complex analyses of family dynamics and children’s cognitive development.

Unique themes and trends in parent–child reading research in the Chinese and English contexts are compared and analyzed. It can be observed that Chinese research emphasizes family responsibility and focuses on the themes of family reading, home-school collaboration, and father participation. Methods such as questionnaires and interviews were employed to investigate the reading conditions in rural areas and the role of fathers in parent–child reading, with a primary focus on the practice of parent–child reading itself. English-context research, rooted in individualistic cultural traditions, employs experimental methods to address reading rights for low-income groups and ethnic minorities, as well as professional interventions in maternal roles, emphasizing the in-depth construction of theoretical frameworks. Additionally, the behavioral differences between the two profoundly reflect the systemic shaping of parental reading behaviors by sociocultural logic. Within China’s context, parents predominantly assume the role of structured guides. Through goal-oriented interactions and picture book materials, they focus on cultivating children’s rule-following, knowledge acquisition, and collective adaptation behaviors, thereby preparing them for behavioral norms required for school entry. In English contexts that prioritize individualism and child-centered approaches, parents tend to adopt roles as emotional supporters and potential facilitators. Through open dialog and materials like wordless picture books, they encourage children to engage in self-expression, imaginative construction, and emotional exploration. This approach significantly fosters the development of creative thinking, autonomy, and social-emotional skills. These two pathways reveal how micro-level interactions transmit macro-level cultural values, leading to distinct developmental focuses for children.

In summary, this research systematically reveals the common characteristics and cultural specificity of parent–child reading research in Chinese and English contexts through quantitative and qualitative analyses and explains the causes of behavioral differences from the perspective of policy context and social values, providing theoretical references for subsequent cross-cultural comparative studies and practical interventions.

## 6. Limitations and Future Research Direction

While the present study offers a systematic mapping of the convergences and divergences between Chinese and English parent–child shared reading research, several methodological and conceptual limitations must be acknowledged. First, the data collection was restricted to peer-reviewed literature indexed in the CNKI and the WOS due to the accessibility of the datasets. The exclusion of other research datasets, such as educational full text, and non-peer-reviewed scholarly works, such as doctoral dissertations, monographs, and policy reports, may have constrained the sample’s representativeness and omitted emerging scholarly works. Second, while CiteSpace is a robust tool for identifying macro-level thematic trends and intellectual turning points, bibliometric co-occurrence analysis is inherently limited in its ability to capture the qualitative nuances and the complexities of reading practices within diverse cultural contexts. Furthermore, the interpretation of cross-cultural data is inevitably subject to the researcher’s own socio-cultural positionality, which introduces a potential risk of interpretive bias when deconstructing values like “autonomy” or “moral cultivation”.

To address these gaps, future research should move beyond theoretical synthesis toward more critical analyses of how researchers in the Chinese and English contexts operationalize parent–child book reading interactions. For example, researchers may scrutinize how studies defined and assessed the quality of parent–child shared book reading and what specific reading behaviors previous studies focused on. In addition, by employing mixed-methods designs that triangulate bibliometric trends with ethnographic observations, future studies can conduct interviews with parents, educators, or researchers in their respective cultural contexts and develop a more holistic and culturally responsive understanding of how shared reading functions as a fundamental scaffold for child development. Furthermore, future research may also examine changes in other related topics to better understand whether parent–child shared reading behavior has evolved alongside social development.

## Figures and Tables

**Table 1:** The 11 keywords with the highest centrality in parent–child reading research in the Chinese and English context.

WOS			CNKI		
Keywords	Frequency	Centrality	Keywords	Frequency	Centrality
Intervention	68	0.15	亲子阅读 Parent–Child Reading	116	1.01
Acquisition	29	0.15	幼儿 Young Children	41	0.29
Family	34	0.14	早期阅读 Early Reading	40	0.25
Emergent Literacy	43	0.13	学前儿童 Preschool Children	20	0.19
Children	41	0.12	亲子共读 Parent–Child Shared Reading	24	0.12
Language	97	0.11	绘本阅读 Picture Reading	16	0.10
Home	43	0.11	农村 Rural	11	0.10
Home Literacy	27	0.10	现状调查 Status Survey	12	0.07
Attachment	12	0.10	影响因素 Influencing Factor	7	0.07
Language Development	35	0.09	儿童阅读 Children’s Reading	4	0.07
Achievement	24	0.08	指导策略 Guiding Strategy	15	0.05

## Data Availability

The dataset used in this research consists of 372 papers from CNKI and 355 from WOS between 2005 and 2024. The data were preprocessed and analyzed using CiteSpace (version 6.3.R3). The raw data is available from the corresponding author upon request.

## Abbreviations

The following abbreviations are used in this manuscript:

CNKI	China National Knowledge Infrastructure
WOS	Web of Science
